# A comparison of two gene regions for assessing community composition of eukaryotic marine microalgae from coastal ecosystems

**DOI:** 10.1038/s41598-024-56993-4

**Published:** 2024-03-18

**Authors:** Jacqui Stuart, Ken G. Ryan, John K. Pearman, Jacob Thomson-Laing, Hannah G. Hampton, Kirsty F. Smith

**Affiliations:** 1 School of Biological Sciences, Victoria University of Wellington, PO Box 600, Wellington, 6140 New Zealand; 2 Cawthron Institute, Private Bag 2, Nelson, 7042 New Zealand

**Keywords:** Ecology, Molecular biology

## Abstract

Two gene regions commonly used to characterise the diversity of eukaryotic communities using metabarcoding are the 18S ribosomal DNA V4 and V9 gene regions. We assessed the effectiveness of these two regions for characterising diverisity of coastal eukaryotic microalgae communities (EMCs) from tropical and temperate sites. We binned amplicon sequence variants (ASVs) into the high level taxonomic groups: dinoflagellates, pennate diatoms, radial centric diatoms, polar centric diatoms, chlorophytes, haptophytes and ‘other microalgae’. When V4 and V9 generated ASV abundances were compared, the V9 region generated a higher number of raw reads, captured more diversity from all high level taxonomic groups and was more closely aligned with the community composition determined using light microscopy. The V4 region did resolve more ASVs to a deeper taxonomic resolution within the dinoflagellates, but did not effectively resolve other major taxonomic divisions. When characterising these communities via metabarcoding, the use of multiple gene regions is recommended, but the V9 gene region can be used in isolation to provide high-level community biodiversity to reflect relative abundances within groups. This approach reduces the cost of sequencing multiple gene regions whilst still providing important baseline ecosystem function information.

## Introduction

Eukaryotic marine microalgae are widespread and diverse microorganisms that occur in many aquatic ecosystems^1^. As the basal food source in the marine food web, their community structure directly influences all life in the ocean [e.g., Refs.^2–4^]. In addition, they are vital participants in key global biogeochemical cycles, including sequestering greenhouse gasses^5^, fixing nitrogen^5,6^, producing atmospheric oxygen^7^ and recycling nutrients^8–10^. Even though they are vital to all ecosystems, there is still a lack of baseline diversity and community structure data on many marine eukaryotic microalgal communities (EMCs) and the ability to easily assess these.

Morphological identification techniques (e.g. microscopic identification and flow cytometry) are effective methods to assess overarching species or community composition but can be extremely time consuming when assessing many taxa with sizes spanning multiple orders of magnitude^11–13^. In addition, when examining community composition, morphological identification lacks sensitivity especially for cryptic microalgae, which contribute an important reservoir of diversity^14^ and the depth of knowledge required for effective taxonomic identification takes a long time to develop^15^. The development of DNA metabarcoding, which utilizes high-throughput sequencing and high quality sequence databases like PR2^16^, has enabled rapid characterisation of eukaryotic communities (EC)^17–21^ and the establishment of baseline datasets for EMCs at a large scale^13,22,23^.

**Figure 1:**
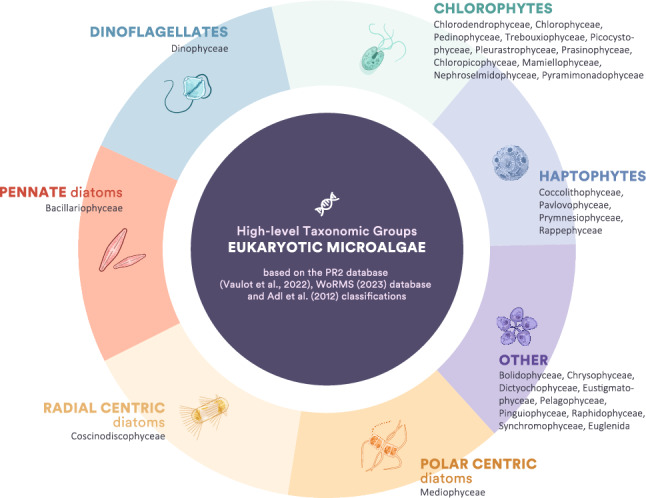
The high-level taxonomic groups of eukaryotic marine microalgae used in this study. The other categories include golden, yellow, and brown classes. Divisions are based on PR2 database classifications (Vaulot et al., 2022), as proposed by Adl et al., (2012) and the World Register of Marine Species (WoRMS 2023) database.

It is important to consider what depth of taxonomic resolution is used when assessing EMC structure, such as species specific or high-level taxonomic group. Division into higher taxonomic groups (Fig. 1) can facilitate a better understanding of their response to different environmental factors and prediction of future states^24–28^. This approach eliminates the need to assess the impact of global change on individual species by focusing on the response of specific taxonomic groups to major environmental shifts^25,29^. It also removes the necessity for databases to fully resolve taxa to species level, which is one of the main limitations of metabarcoding when used to characterise EMCs. Gene region and primer selection can also bias the diversity observed in communities when using metabarcoding approaches and requires careful consideration^30–32^.

The use of environmental DNA (eDNA) and metabarcoding for characterising EMC composition and diversity are quickly becoming standard approaches, with research focus shifting to optimisation of techniques, including the assessment of sampling methods^33,34^, improvement in preservation and extraction techniques^35,36^, and development of bioinformatic pipelines^37,38^. Selection of gene regions, primers and understanding of the bias they introduce are important considerations when using molecular techniques^39^, with taxonomic resolution and the availability of reference sequences also influencing results^16,40^. Upward of 12 gene regions are commonly used to characterise microalgal communities, dependent on the taxonomic group of interest^41^, with a combination of gene regions commonly used or suggested to overcome bias of any single gene region/primer pair^21,42,43^. Some of the most used regions include the 18S ribosomal DNA (rDNA) V4 and 18S rDNA V9^41^.

Comparisons of the 18S rDNA V4 and V9 regions, and their coverage of diversity within EMCs has been assessed^30–32^. However, the focus is usually on the depth of taxonomic resolution and total diversity observed. In addition, comparison of EMCs in temperate and tropical zones, or comparing two or more biomes in general, have been previously undertaken, though most on single taxon as opposed to the broader microalgae community [e.g., Ref.^13^,^45^]. To date, there have been few comparison of the community composition of marine EMCs via metabarcoding of the V4 and V9 regions alongside microscopic analyses, that focus entirely on the eukaryotic microalgae community at a higher taxonomic level. The use of multiple gene regions can be a comprehensive approach [e.g., Ref.^42^], however this is not always possible within budget restrictions.

This study compares the effectiveness of the two commonly used variable regions (V4 and V9) of 18S rDNA for metabarcoding in characterizing community composition and the underlying diversity of EMCs. Tropical and temperate coastal sites were selected to assess the structure of the EMC using both metabarcoding and microscopic identification. This approach aims to determine the rDNA region that aligns best with observed communities at the high-level taxonomic group level. The results will help streamline large-scale community assessments of EMCs, fill vital data gaps, and reduce the cost of biodiversity assessments. A better understanding of the current state of these communities will enhance our comprehension of their response to changing climatic and environmental conditions in the future.

## Results

Illumina MiSeq sequencing for the broader eukaryotic community (EC) from the temperate site provided a total of 907,560 and 2965,681 processed reads for the 18S rDNA V4 and V9 regions respectively. Of the total reads that were processed into ASVs, the EMC made up 17% of the V4, and 21% of the V9 results. Total processed reads from sequencing at the tropical site were 938,681 (V4) and 1,728,490 (V9). The tropical EMC represented 30% of the V4 EC reads that were processed into ASVs and 31% of the V9 EC. The V4 region detected lower numbers of all taxonomic levels than the V9 for both sites. Around twice as many orders and families were detected by the V9 region, and more genera and species by ≥ 20×. Taxa that could not be classified lower than class level for the V4 region were mostly dinoflagellates, with all other unidentified taxa from the other high level taxonomic groups contributing ≤ 1% of V4 ASVs. This was consistent across both sites (Table 1). Dinoflagellates also made up the largest proportion taxa not classified below the class level for the V9 region. Additionally, pennate diatoms made up a higher portion of unclassified (class level) ASVs, especially at the temperate site. Overall, the V4 region only outperformed the V9 consistently at species level.

**Table 1:** Detection and sequencing resolution across taxonomic levels for the 18S ribosomal DNA V4 and V9 region amplicon sequence variants (ASVs) from tropical and temperate site eukaryotic microalgae community (EMC).

Site	Region	Order (ASV %)	Family (ASV %)	Genus (ASV %)	Species (ASV %)	Taxa not able to be classified past Class level
Temperate	V4	20 (71%)	34 (63%)	46 (56%)	67 (42%)	Dinoflagellates 28% All other groups ≤ 1%
Temperate	V9	45 (78%)	66 (67%)	85 (59%)	107 (38%)	Dinoflagellates 12% Pennate Diatoms 3% Polar Centric Diatoms 1% Radial Centric Diatoms 1% Haptophytes 2% Other microalgae 3%
Tropical	V4	14 (74%)	29 (71%)	48 (62%)	68 (49%)	Dinoflagellates 28% Other groups ≤ 1%
Tropical	V9	41 (62%)	51 (52%)	68 (45%)	90 (31%)	Dinoflagellates (21%) Pennate diatoms (15%) All other groups ≤ 1%

Results are presented as the number of taxa detected at each level, followed in brackets with the percent of ASVs positively identified at that taxonomic resolution.

**Figure 2:**
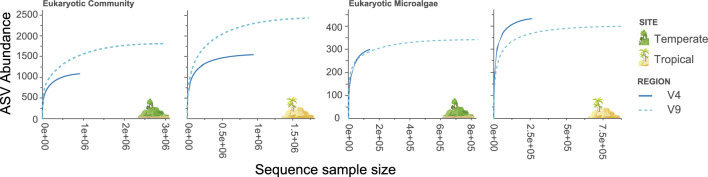
Diversity rarefaction curves of the 18S ribosomal DNA V4 (solid line) and V9 (dotted line) amplicon sequence variants (ASVs) from the entire eukaryotic community and subset of the eukaryotic microalgal community at the temperate and tropical sites.

Rarefaction analysis was conducted on both the complete EC and an EMC subset at both sites to assess coverage of ASVs. Curves for the EC for both gene regions at the temperate and tropical sites reached a plateau indicating ASV diversity was saturated, with the number of ASVs greater for the V9 region at both sites (Fig. 2). When assessing rarefaction for the EMC, curve saturation for both gene regions at the tropical sites and the V4 region from temperate site showed that sequencing effort was sufficient to assess total ASV diversity (Fig. 2). The temperate V9 region was a bit low but was still assessed.

### Diversity indices

**Figure 3:**
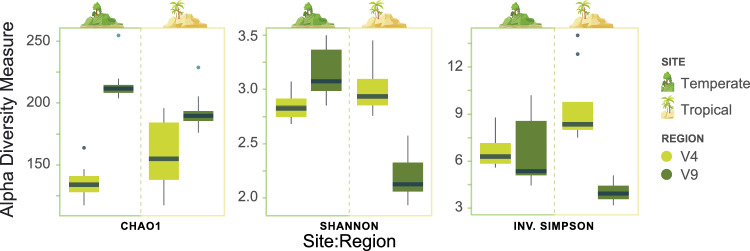
Alpha diversity indices for the eukaryotic microalgae community (EMC) at temperate and tropical sites, comparing Amplicon sequence variants (ASV) data of the 18S ribosomal DNA (rDNA) V4 and V9 region. Diversity indices include Chao1, Shannon and Inverse Simpson. All V4 and V9 samples were rarefied to a sequence depth of 11000.

Metabarcoding read data was rarefied to an even depth of 11000 for direct comparison of the alpha diversity indices between the V4 and V9 regions across both sites (Fig. 3; Supplementary Table S7). Chao1, Shannon and Inverse Simpson indices were selected to assess richness (with sensitivity to rare ASVs), evenness and diversity accounting for both richness and evenness at each site. The Shannon and Inverse Simpson indices showed higher values at the tropical site than the temperate according to the V4 region, with the v9 showing the opposite pattern. Chao1 values (species richness) for the V4 region were lower than the V9 region at both sites, indicating the V4 region captured less of the species richness at each site (pairwise Wilcox test (PW): temperate p = 6e − 04; tropical: p = 4.7e − 03). Shannon indices values show the V9 captured more diversity than the V4 at the temperate site (PW: p = 0.015), however at the tropical site the V4 region captured more of the diversity than the V9 (PW: p = 3.73e − 04). The inverse Simpson indices showed a higher median value for the V4 region at both sites (PW: temperate p = 0.39; tropical p = 4e − 04) with the disparity between captured diversity far higher on the tropical site, a trend also seen with the Shannon indices (Fig. 3).

### EMC composition

**Figure 4:**
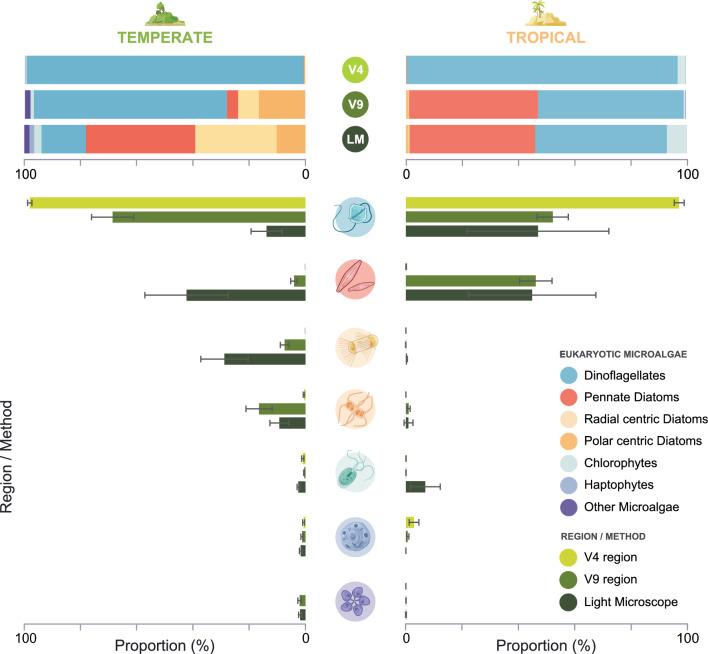
Eukaryotic microalgal taxonomic groups retrieved using metabarcoding of the 18S ribosomal DNA (rDNA) V4 and V9 regions, and light microscopy cell count data (LM) from a) temperate, and b) tropical sites. Eukaryotic microalgae are divided into high-level taxonomic groups as defined in methods section *Bioinformatic analysis.*

Commuinity composition was variable among sites, gene regions and cell counts, however the proportional abundance from metabarcoding of the V9 region EMC more consistently aligned to light microscopy (LM) observations than the V4 region (Fig. 4). At both sites the V4 region proportional abundance of high level taxonomic groups showed dinoflagellate dominant communities (97—98%: Fig. 4) with very little contribution from any other groups. Dinoflagellates also proportionally made up the majority of the EMC based on the V9 region with 69% and 52% at temperate and tropical sites respectively. Light microscopy based EMC observed a substantial difference in dominant groups at the temperate site for both V4 and V9, with dinoflagellates only contributing 14%. Observations for LM from the tropical site did not show much deviation from the V9 metabarcoding results, dinoflagellates made up 47% of the community. High-level taxonomic groups had significant variation in detection success between gene regions at both sites. At the temperate site, LM showed pennate, radial centric and polar centric diatoms made substantial contributions to the EMC community. Propotional abundance for the V9 region had smaller contributions from the pennate and radial centric diatoms compared to LM, and a higher proportion of polar centric. In the V4 all groups had negligible proportions, with dinoflagellates dominating the community.

**Figure 5:**
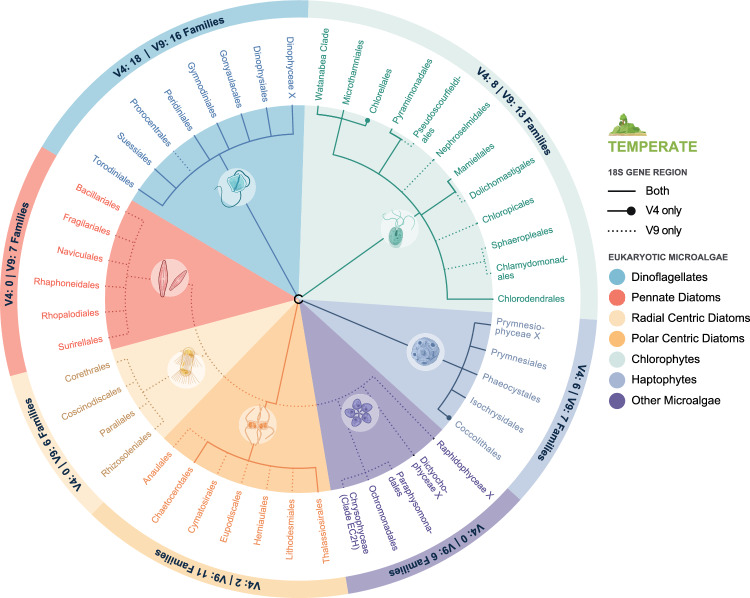
Total eukaryotic microalgae detected in environmental DNA (eDNA) extracted from 27 samples collected at the temperate site using metabarcoding from the 18S V4 and V9 regions analysed at the Order level. Each pie slice represents eukaryotic microalgal taxonomic groups, including dinoflagellates, chlorophytes, haptophytes, pennate diatoms, radial centric diatoms, polar centric diatoms, and other eukaryotic microalgae. External nodes of the dendrogram represent microalgal orders in each taxonomic group. In addition, the annotations within the external circle show the number of families detected within each of the overarching taxonomic groups.

When assessing taxonomic divisions at order level from metabarcoding data the V9 region identified 27 and 30 more taxa than the V4, at the temperate and tropical sites respectively. Therefore, unsurprisingly at the family level the V9 detected over twice the number of taxa compared to the V4 region. The temperate site showed the occurrence of six pennate diatom orders (Bacillariales, Fragilariales, Naviculales, Rhaphoneidales and Surirellales) and four radial centric orders (Corethrales, Cosinodiscales, Paraliales and Rhizosoleniales), all only detected by the V9 region (Fig. 5). Additionally, only two of the seven polar centric classes (Anaulales, Chaetocerotales, Cymatosirales, Eupodiscales, Hemiaulales, Lithodesmiales and Thalassiosirales) were observed using the V4 region, all of which were detected by the V9 region. Dinoflagellates in Orders Dinophysiales, Gonyaulacales, Gymnodiniales, Peridiniales, Prorocentrales, Suessiales and Torodiniales were also detected at the temperate site*.* Both regions detected six of the seven classes, however the Order Prorocentrales was only detected with V9. Chlorophytes were also more readily detected by the V9 region. Twelve chlorophyte orders were observed in total with both regions detecting five (Chlorodendrales, Mamiellales, Pyramimonadales, Microthamniales and Watanabea Clade). However, the V9 detected an additional six orders (Chlamydomonadales, Sphaeropleales, Chloropocales, Dolichomastigales, Nephroselmidales and Pseudoscourfieldiales), and the V4 one additional order (Chlorellales). Haptophyte orders Prymnesiales, Phaeocystales and Isochrysidales were detected using both gene regions with the addition of Coccolithiales detected only by the V4. Orders falling under the ‘other’ category were only detected using the V9 region and included Chrysophyceae (Clade EC2H), Ochromonodales and Paraphysomonadales. In addition, at the family taxonomic level, the V4 region was less sensitive, consistently detecting fewer families for all high-level taxonomic groups, excluding dinoflagellates. More ASVs were identified to species level by the V4 region (69) than the V9 (45) for dinoflagellates at this site (Supplementary Table S1).

**Figure 6:**
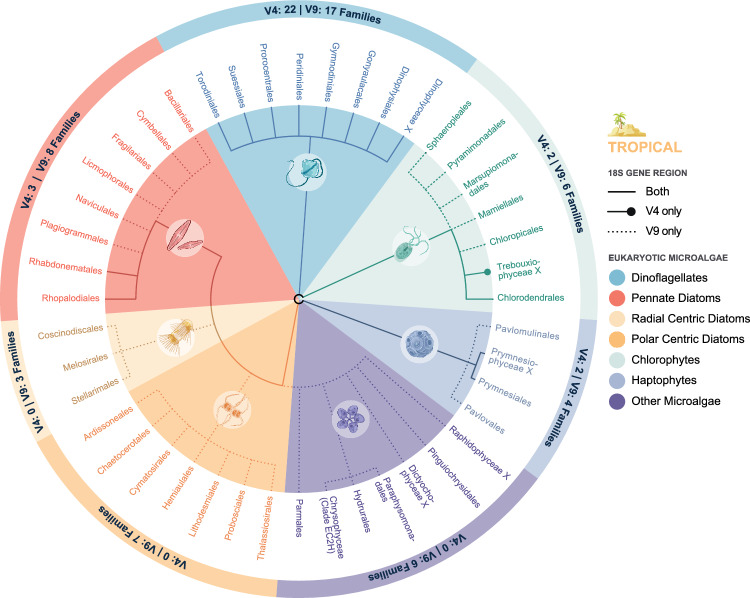
Total eukaryotic microalgae detected in environmental DNA (eDNA) extracted from 27 samples collected at the tropical site using metabarcoding from the 18S rDNA V4 and V9 regions analysed at the Order level. Each pie slice represents eukaryotic microalgal high-level taxonomic groups, including dinoflagellates, chlorophytes, haptophytes, pennate diatoms, radial centric diatoms, polar centric diatoms, and other eukaryotic microalgae. External nodes represent microalgal orders in each group and the outer circle notes Families detected within each high-level taxonomic group.

At the tropical site eight pennate diatoms orders were detected (Bacillariales, Cymbellales, Fragilariales, Licomophorales, Naviculales, Plagiogrammales, Rhabdonematales and Rhopalodiales), with only Rhabdonematales and Rhopalodiales detected by V4 region (Fig. 6). A total of eight dinoflagellate classes were detected by both the V4 and V9 regions. A single Chlorophyte order was detected by both regions (Chlorodendrales), an additional one by the V4 region (Trebouxiophyceae) and a further five by the V9 region only (Chloropicales, Mamiellales, Marsupiomonadales, Pyramimonadales and Sphaeropleales). Within the Haptophyte group four orders were detected in total, with one by both regions (Prymnesiales), and two by V9 only (Pavlomulinales and Pavlovales). As at the temperate site, all orders from the ‘other’ category were only detected by the V9 region. Additionally, when assessing detected diversity at family level the V4 was again less sensitive to all high-level taxonomic groups than the V9 region, apart from dinoflagellates. Almost twice as many dinoflagellate ASVs at the tropical site were successful identified to species level by the V4 region (143) compared to the V9 (73; Supplementary Table S2).

## Discussion

We assessed the effectiveness of the 18S rDNA V4 and V9 gene regions to characterise coastal marine EMCs. Each of the gene regions showed substantial differences in high throughput sequencing efficiency, ASV numbers, diversity measures, relative abundance, and taxonomic resolution. The V9 region generated more raw reads than the V4 at both sites. This aligns with other studies assessing both the EC^42^ and EMC, where up to twice as many raw reads were produced by the V9^30,42^. Greater raw reads generated when using V9 are most likely a product of the shorter fragment length for this gene region. As a result, for the same sequencing effort (e.g., same number of samples per sequencing plate) more reads are produced on Illumina MiSeq for V9. This would potentially allow for the V9 gene region to capture more diversity present within the sites, especially those organisms that occurred in low abundance^46^. However, rarefaction analysis indicated that both gene regions reached saturation and thus a good estimation of the diversity of community detected by each region should have been achieved.

Higher proportions of V4 ASVs were successfully assigned at the species levels compared to the V9 gene region. Interestingly, this greater depth of taxonomic resolution was not aligned with captured diversity overall and indicated a clear bias to dinoflagellates over other taxonomic groups. Higher taxonomic resolution achieved using the V4 marker for dinoflagellates may be a consequence of high sequence variation of dinoflagellates within the V4 region^41^, or the greater length of this region increasing the chances of species level nucleotide variation being captured. Dinoflagellates have been shown to have very high rDNA copy number with a lot of variation even within a strain^47,48^. This bias to dinoflagellates may also influence the higher levels of diversity indicated by the Shannon and inverse Simpson indices for the V4 region compared to the Chao1. The latter accounts mainly for richness, or the number of taxonomic groups in the ecological community, while the Shannon and inverse Simpson, incorporate species evenness, or the distribution and the abundance of the taxonomic groups^49^. With a greater number of dinoflagellate ASVs identified by the V4 region at both sites it is clear the indices are not accounting for the bias of captured diversity. This highlights potential limitations of these ecological diversity indices when using metabarcoding data, where more ASVs do not necessarily represent diversity of all taxonomic groups in a community evenly.

Comparison of the proportional abundance of each gene region to LM community composition showed the V9 was more representative of the LM observed community. Unlike communities observed by Stoeck^44^, V4 and V9 taxonomic profiles were distinctly different with entire high level taxonomic groups being consistently missed by the V4 primers. Results from the V4 region at both sites were monopolised by dinoflagellates ASVs and only a very small portion of the community was made up of diatoms or chlorophytes. Light microscopy observations for the tropical site were very closely aligned to the V9, while the temperate site had the most substantial variation of dinoflagellates between each gene region. Again, this could be related to the influence of dinoflagellate cell size on gene copy number, where larger cells tend to have more rDNA gene copies^48^, or intra-cellular variation of the rDNA gene regions detected as multiple ASVs. Previous studies have shown that community composition based on LM generally vary greatly in comparison to next-generation sequencing or metabarcoding^41^, with both more and less diversity reported^50–52^. Identification of EMC taxa by morphology is complicated, requiring time, effort, and often experts specialised in specific taxonomic groups to confidently assign specimens to genus or species level. In under-studied environments where the species composition is less characterised, the chances of misidentification at the genus/species levels increases. Thus, undertaking EMC surveys at higher taxonomic levels for both metabarcoding and LM would provide essential baseline data. This can enable greater understanding of high-level ecosystem function, in addition to reducing time when comparing metabarcoding data with LM.

Lack of detection of the majority of diatom and ‘other’ microalgal taxa by the V4 could indicate a deficit in the databases used. It is widely acknowledged that the number of sequences for V4 and V9 regions differ across open-source sequence databases [Refs.^30^,^46^]. Primer bias could also contribute to this, though interestingly, the V4 was identified by Hadziavdic, Lekang^53^ along with the 18S V2 and V9 as being best suited for biodiversity assessments due to the quality of universal primers. A limitation of metabarcoding for microalgal communities in general is that many of the databases are restricted to sequences from isolates that can be cultured. Cultured isolates provide clean and high-quality reference sequences linked to taxonomic information, however strains that are hard to isolate or do not do well in culture conditions are underrepresented in all genetic databases. Therefore, all EMC surveys will most likely be mis-representing the community to a degree.

This comparison of LM and metabarcoding to assess and characterise diversity in the EMC showed the 18S rDNA V9 region to be a closer representation of LM observed community than the 18S rDNA V4 region. Additionally, the V9 captures more of the community diversity at higher taxonomic classification, including microalgal groups present in low abundance. If the aim is to achieve a high-level understanding of the ecosystem dynamics in relation to the EMC, then the use of V9 in isolation can be recommended. For achieving greater taxonomic depth, as recommended in previous literature, the combination of both gene regions or utilising group-specific primers [or haptophytes: Ref.^54^, e.g. dinoflagellates: Ref.^55^] will provide greater coverage. Ground truthing metabarcoding results with cell count data using LM will increase the rigor of the data generated and confidence in metabarcoding assessments. An accurate and robust understanding of eukaryotic microalgal diversity and distribution is vital for filling baseline data gaps that inform on ecosystem function and diversity. This research reinforces that the gene region choice for metabarcoding can have a substantial impact on the accuracy and comprehensiveness of EMC assessments, offering vital insights for improving our understanding of these communities' diversity, dynamics, and ecological roles.

## Methods.

### Sample collection

Samples were collected from one temperate (New Zealand) and one tropical site (Cook Islands) (Table 2). Surface water temperature and salinity at the temperate site averaged 14.8 °C and 31.1 ppt respectively on the day of sampling. The average surface temperature at the tropical site was 25 °C and the salinity was 35.5 ppt on the day of sampling. Both sites were coastal or near shore and had no significant rainfall two to three days prior to sampling. All samples were collected in spring (Table 2). At each site, vertical plankton net tows starting at 5 m depth, or from the seabed if the water column was less than five m deep, were completed using a weighted 15–20-micron phytoplankton net. Nine sampling points were selected within each site, with three net tow replicates completed at each (n = 27). Around 200 L of water was filtered through the net for each replicate tow. Samples were placed into 500 ml plastic containers for transport back to shore, 40 mL of each were transferred to 50 mL falcon tubes for cell counts, and 2 mL of the preservation solution lugols was added. The remainder of each sample (100–200 mL) was then filtered within three hours of collection through 0.45 µm Durapore® PVDF Membrane filter (Sigma-Aldrich). Filters were fully submerged in a nucleic acid preservative (RNAlater; ThermoFisher Scientific, USA) in 2.5 mL Eppendorf tubes for approx. 24 h and then stored at – 20 °C until DNA extraction.

**Table 2:** Site information, including sampling date, GPS co-ordinates, sampling location, sampling points (Points), replicates per site (Rep.), total samples per site (n) and environmental DNA sampling method.

Date	Co-ordinates	Location	Points	Rep	n	ecoregion
20.10.2021	− 41.218, 173.094	Nelson Tasman, New Zealand	9	3	27	Temperate
28.11.2022	− 21.274, − 159.743	Rarotonga, Cook Islands	9	3	27	Tropical

### DNA Extraction

DNA was extracted from all environmental samples using the DNeasy PowerSoil Pro kit (QIAGEN, California, USA) following the manufacturers protocol. This included for both the tropical and temperate sites, three replicate samples taken at nine sampling point (n = 27 per site). Briefly, each sample filter was removed from the RNA-Later preservative and placed directly into the bead tube provided with the extraction kit, the remaining preservation solution was then centrifuged (1 min, 3,000 g). The resulting pellet was also added to the bead tube to maximise capture of any material that shed from filters during storage. Samples were homogenised for 2.5 min, centrifuged again, then extraction protocols were completed using a Qiagen QIACube robot (Qiagen, Carlsbad, USA). Quantificaition of extracted DNA concentrations was undertaken using the NanoPhotometer® NP80 (Implen Inc. Munich, Germany), tropical samples ranged from 5.1 and 11.2 ng/µL and temperate samples from 24.4 and 59.4 ng/µL (Supplementary table S3).

### Polymerase chain reaction amplification and sequencing

The 18S rDNA V4 region (270 bp–387 bp) was amplified using primer pairs Uni18S-F and Uni18S-R^56^ and the V9 (96 bp–134 bp) region with primer pair 1380-F and 1510-R^57^. Each primer pair was modified for Illumina sequencing with the addition of overhang adaptors (Supplementary Table S4). Polymerase chain reactions (PCRs) contained: 25 µL 2 × MyFi™ Mix (Bioline, UK), 0.4 µM of both forward and reverse primers, and between 5 and 50 ng of template DNA with a total volume of 50 µL. Thermocycling conditions for the V4 and V9 regions were as specified in Supplementary Table S4. Sample amplification was confirmed with visualisation under UV light of 1.5% agarose gels using Red Safe™ Loading Dye (Herogen Biotech, USA). Negative PCR reactions, extraction blanks and water blanks were run alongside samples to check for contamination. Amplified samples were then purified using the SequalPrep™ Normalisation plate (ThermoFisher, MA, USA), and sent to Sequench (Nelson, New Zealand) for library preparation and MiSeq Illumina Sequencing. Samples for both regions were indexed using the Nextera v2 Kit, quality control with Bioanalyzer and quatification with Qubit. Library preparation and sequencing were undertaken using the v3 2 × 300 bp kit for the V4 region and v2 2 × 150 bp kit for the V9.

### Bioinformatic analysis

Raw sequences for each replicate sample (n = 27 per site) and blank samples generated via Illumina MiSeq were trimmed using *cutadapt* (Martin 2011) to remove the primers (allowed mismatch = 1), then further processed with the *DADA2* pipeline^58^. Base pipeline parameters were adjusted as defined in the following section. Sequences were truncated (V9: 128 and 130 bp and V4: 288 and 230 bp) and filtered for maximum “expected errors” (maxEE) of 2 and 4 for forward and reverse reads respectively. Any reads not meeting the defined thresholds were discarded at this point. ASVs were infered based on a parametric error matrix constructed from the first 10^8^ bp. Pair-end amplicon sequences were then merged using maxmismatch = 1 and overlap = 10. The resulting ASVs were checked for chimeras and ASVs outside the expected length of the amplicon were trimmed (Supplementary table S5). ASVs were then classified against the PR2 (v5) database^59^ using rdp^60^ with a minBoot of 70 in DADA2 to enable classification at higher taxonomical levels (Supplementary table S6). Negative controls were assessed for potential contamination. The maximum number of reads observed across the negative controls for each ASV was removed via subtraction from the samples. Rarefaction curves (observed species richness) were produced in R using *ggplot2* (Wickham 2016) and ranacapa^61^ to compare diversity of ASVs across sampling points at each site. Alpha diversity and community composition analysis was undertaken using the *Vegan* package. Diversity indices undertaken included Chao1 to assess richness, Shannon and Inverse Simpson to assess both richness and evenness on each sampling point within a site (n = 9). Each sampling point for both regions were rarefied to an even depth of 11,000 reads for inter-region diveristy comparison.

The community composition of the EMC was divided into taxon-based groups: dinoflagellates (Dinophyceae), pennate diatoms (Bacillariophyceae) radial centric diatoms (Coscinodiscophyceae), polar centric diatoms (Mediophyceae), chlorophytes (Chlorodendrophyceae, chlorophyceae, chloropicophyceae, mamiellophyceae, nephroselmidophyceae, pedinophyceae, picocystophyceae, prasinophyceae, pyramimonadophyceae, trebouxiophyceae), haptophytes (Coccolithophyceae, pavlovophyceae, Prymnesiophyceae, Rappephyceae), and ‘Other’ eukaryotic microalgae (Bolidophyceae, chrysophyceae, dictyochophyceae, eustigmatophyceae, pelagophyceae, pinguiophyceae, raphidophyceae, Synchromophyceae, Euglenida). Taxonomic ranks were identified using the revised classification of eukaryotic groups proposed by Adl, Simpson^62^, which is also used for taxonomic assignment in the PR2 database. This resolution was based on taxon-based divisions used to assess the microalgal community structure [Fig. 1; 24, 25–28]. The full pipeline from this analysis is published on GitHub (https://github.com/JustJaxz/Region-Comparison-Pipeline).

### Community composition using light microscopy

Cell counts were completed using lugol preserved samples to compare to amplicon sequence variant (ASV) relative abundance. An additional plankton net tow was complete at each sampling point (n = 9 per site) and three replicate counts were complete on each of these samples. Identification of eukaryotic microalgae into taxonomy-based groups was undertaken with groups divided as previously defined in Sect. “Bioinformatic Analysis”. Morphological identification was aided with the use of live cultures from the Cawthron Institute Culture Collection of Microalgae (CICCM, Cawthron Institute, Nelson, New Zealand) and published morphological descriptions and guides [e.g., Refs.^63–65^]. Cell counts were completed using Utermöhl chambers holding 10 mL of samples, each settled for a minimum of 12 h. Microscopic transects were complete in triplicate under either 200–400 × magnification, dependant on cell density in samples with a Olympus CKX41 Inverted Phase Contrast Fluorescence Microscope (Olympus Life Sciences, Japan). Cell concentration was calculated and visualised as relative abundance/proportion alongside ASV data using R.

### Supplementary Information

Supplementary Information.

## Data Availability

The raw sequences were submitted to NCBI short read archive under accession number: PRJNA1028654.
