# Supplementary material for: A comparison of two gene regions for assessing community composition of eukaryotic marine microalgae from coastal ecosystems
Source: Sci Rep. 2024 Mar 18;14:6442. doi: 10.1038/s41598-024-56993-4 (PMC10948787; doi:10.1038/s41598-024-56993-4)
Supplement: Supplementary file 1 — Supplementary Information. [file 41598_2024_56993_MOESM1_ESM.pdf]

Supplementary material

A comparison of two gene regions for assessing eukaryotic marine microalgal community composition on coastal sites.

Jacqui Stuart[1, 2], Ken Ryan[1], John Pearman[2], Jacob Thompson-Lang[2], Hannah Hampton[2], Kirsty F. Smith[2, 3]

1) School of Biological Sciences, Victoria University of Wellington, PO Box 600, Wellington 6140, New Zealand. 2) Cawthron Institute, Private Bag 2, Nelson 7042, New Zealand. 3) School of Biological Sciences, University of Auckland, Private Bag 92019, Auckland 1142, New Zealand.

Contents

Supplementary table S1 · · · · · · · · · · · · p2-6

Supplementary table S2 · · · · · · · · · · · · p7-11

Supplementary table S3 · · · · · · · · · · · · p12

Supplementary table S4 · · · · · · · · · · · · p13

Supplementary table S5 · · · · · · · · · · · · p14-15

Supplementary table S6 · · · · · · · · · · · · p16

Supplementary table S7 · · · · · · · · · · · · p16

Supplementary Table S1. List of ASVs identified to species level for temperate site samples (Mckee Reserve, Nelson, New Zealand), detected by the 18s rDNA V4 and V9 gene regions.

GREEN ALGAE

Phylum	*class*	*order*	*family*	*genus*	*species*	*region*	*ASV v4*	*ASV v9*
Chlorophyta	Chlorodendrophyceae	Chlorodendrales	Chlorodendraceae	Tetraselmis	*Tetraselmis convolutae*	v4	2	-
	Chlorophyceae	Chlamydomonadales	Chlamydomonadales X	Chlamydomonas	*Chlamydomonas parkeae*	v9	-	1
		Sphaeropleales	Sphaeropleales X	Tetradesmus	*Tetradesmus arenicola*	v9	-	1
	Chloropicophyceae	Chloropicales	Chloropicaceae	Chloroparvula	*Chloroparvula pacifica*	v9	-	1
					Chloropicon roscoffensis	v9	-	1
					Chloropicon sieburthii	v9	-	1
	Mamiellophyceae	Mamiellales	Bathycoccaceae	Bathycoccus	*Bathycoccus prasinos*	v9	-	1
				Ostreococcus	*Ostreococcus tauri*	v9	-	1
			Mamiellaceae	Micromonas	*Micromonas bravo*	v4, v9	1	1
					Micromonas pusilla	v4	1	-
	Nephroselmidophyceae	Nephroselmidales	Nephroselmidales X	Nephroselmis	*Nephroselmis pyriformis*	v9	-	3
	Pyramimonadophyceae	Pyramimonadales	Pyramimonadales X	Cymbomonas	*Cymbomonas tetramitiformis*	v4, v9	2	3
		Pseudoscourfieldiales	Pycnococcaceae	Pseudoscourfieldia	*Pseudoscourfieldia marina*	v9	-	2
		Pyramimonadales	Pterospermaceae	Pterosperma	*Pterosperma cristatum*	v4	1	-
			Pyramimonadaceae	Pyramimonas	*Pyramimonas australis*	v4	1	-
					Pyramimonas disomata	v9	-	2
					Pyramimonas tetrarhynchus	v4	1	-
	Trebouxiophyceae	Watanabea-Clade	Watanabea-Clade X	Apatococcus	*Apatococcus lobatus*	v9	-	1
				Diplosphaera	*Diplosphaera chodatii*	v4	1	-
				Heterochlorella	*Heterochlorella luteoviridis*	v4	1	-

DINOFLAGELLATES

Phylum	*class*	*order*	*family*	*genus*	*species*	*region*	*ASV v4*	*ASV v9*
Dinoflagellata	Dinophyceae	Dinophyceae X	Dinophyceae XX	Abedinium	*Abedinium dasypus*	v4, v9	4	1
		Dinophysiales	Dinophysiaceae	Dinophysis	*Dinophysis acuminata*	v4, v9	1	1
					Dinophysis acuta	v9	-	1
			Oxyphysiaceae	Phalachroma	*Phalacroma porodictyum*	v4	1	-
		Gonyaulacales	Pyrocystaceae	Alexandrium	*Alexandrium fraterculus*	v9	-	2
					Alexandrium insuetum	v4, v9	2	1
					Alexandrium margalefii	v9	-	1
					Alexandrium ostenfeldii	v4	1	-

Phylum	***class***	***order***	***family***	***genus***	***species***	***region***	***ASV v4***	***ASV v9***
Dinoflagellata	Dinophyceae	Gonyaulacales	Pyrocystaceae	Alexandrium	*Alexandrium pacificum*	v4, v9	3	2
			Gonyaulacaceae	Gonyaulax	*Gonyaulax polygramma*	v4, v9	1	1
					Gonyaulax spinifera	v4, v9	8	4
				Spiniferites	*Spiniferites mirabilis*	v9	-	1
					Spiniferites ramosus	v9	-	1
			Ceratiaceae	Tripos	*Tripos furca*	v4, v9	1	1
					Tripos fusus	v4, v9	1	1
					Tripos tenuis	v9	-	3
		Gymnodiniales	Gymnodiniaceae	Akashiwo	*Akashiwo sanguinea*	v9	-	1
				Gymnodinium	*Gymnodinium microreticulatum*	v4, v9	1	3
				Gyrodinium	*Gyrodinium dominans*	v4	1	-
					Gyrodinium fusiforme	v4	1	-
					Gyrodinium gutrula	v9	-	1
					Gyrodinium helveticum	v4	2	-
			Kareniaceae	Karenia	*Karenia brevis*	v4	2	-
				Karlodinium	*Karlodinium veneficum*	v9	-	1
			Gymnodiniaceae	Lepidodinium	*Lepidodinium chlorophorum*	v4	1	-
					Lepidodinium viride	v9	-	1
				Margalefidinium	*Margalefidinium fulvescens*	v9	-	1
				Polykrikos	*Polykrikos geminatus*	v9	-	1
					Polykrikos kofoidii	v4, v9	3	1
				Togula	*Togula britannica*	v9	-	1
		Peridiniales	Amphidiniopsidaceae	Archaeperidinium	*Archaeperidinium minutum*	v9	-	1
			Diplopsalidaceae	Diplopsalis	*Diplopsalis caspica*	v4	1	-
				Gotoius	*Gotoius excentricus*	v4	1	-
			Thoracosphaeraceae	Ensiculifera	*Ensiculifera mexicana*	v4	1	-
Dinoflagellata	Dinophyceae	Peridiniales	Heterocapsaceae	Heterocapsa	*Heterocapsa nei/rotundata*	v4	4	-
					Heterocapsa niei	v9	-	1
			Amphidiniopsidaceae	Islandinium	*Islandinium tricingulatum*	v4, v9	2	1
			Protoperidiniaceae	Protoperidinium	*Protoperidinium bipes*	v4	2	-
					Protoperidinium claudicans	v4	4	-
					Protoperidinium conicum	v4	1	-
					Protoperidinium denticulatum	v4	3	-
					Protoperidinium elegans	v4	4	-
					Protoperidinium fusiforme	v9	-	1

Phylum	*class*	*order*	*family*	*genus*	*species*	*region*	*ASV v4*	*ASV v9*
Dinoflagellata	Dinophyceae	Peridiniales	Protoperidiniaceae	Protoperidinium	*Protoperidinium pellucidum*	v4, v9	5	1
					Protoperidinium punctulatum	v4	1	-
					Protoperidinium thulesense	v9	-	1
			Thoracosphaeraceae	Scrippsiella	*Scrippsiella erinaceus*	v9	-	1
		Prorocentrales	Prorocentraceae	Prorocentrum	*Prorocentrum cordatum*	v9	-	1
		Suessiales	Suessiaceae	Ansanella	*Ansanella granifera*	v9	-	1
				Biecheleria	*Biecheleria cincta*	v4	2	-
				Dactylodinium	*Dactylodinium arachnoides*	v4, v9	1	1
				Pelagodinium	*Pelagodinium beii*	v9	-	3
		Torodiniales	Torodiniaceae	Torodinium	*Torodinium robustum*	v4	3	-

PENNATE DIATOMS

Phylum	*class*	*order*	*family*	*genus*	*species*	*region*	*ASV v4*	*ASV v9*
Gyrista	Bacillariophyceae	Bacillariales	Bacillariaceae	Pseudo-nitzschia	*Pseudo-nitzschia delicatissima*	v9	-	1
					Pseudo-nitzschia fraudulenta	v9	-	1
					Pseudo-nitzschia heimii	v9	-	1
					Pseudo-nitzschia multiseries	v9	-	1
		Fragilariales	Fragilariaceae	Grammonema	*Grammonema striatula*	v9	-	1
		Naviculales	Pleurosigmataceae	Pleurosigma	*Pleurosigma intermedium*	v9	-	1
		Rhaphoneidales	Asterionellopsidaceae	Asteroplanus	*Asteroplanus karianus*	v9	-	1
		Rhopalodiales	Rhopalodiaceae	Epithemia	*Epithemia pelagica*	v9	-	1

RADIAL CENTRIC DIATOMS

Phylum	*class*	*order*	*family*	*genus*	*species*	*region*	*ASV v4*	*ASV v9*
Gyrista	Coscinodiscophyceae	Corethrales	Corethraceae	Corethron	*Corethron hystrix*	v9	-	1
		Coscinodiscales	Coscinodiscaceae	Coscinodiscus	*Coscinodiscus radiatus*	v9	-	1
					Coscinodiscus wailesii	v9	-	1
			Heliopeltaceae	Actinoptychus	*Actinoptychus sinensis*	v9	-	1
			Hemidiscaceae	Actinocyclus	*Actinocyclus curvatulus*	v9	-	1
		Paraliales	Paraliaceae	Paralia	*Paralia sulcata*	v9	-	1
		Rhizosoleniales	Rhizosoleniaceae	Guinardia	*Guinardia flaccida*	v9	-	1
				Rhizosolenia	*Rhizosolenia similoides*	v9	-	1

POLAR CENTRIC DIATOMS								
Phylum	***class***	***order***	***family***	***genus***	***species***	***region***	***ASV v4***	***ASV v9***
Gyrista	Mediophyceae	Anaulales	Anaulaceae	Ceratanaulus	*Ceratanaulus creticus*	v9	-	1
		Chaetocerotales	Chaetocerotaceae	Chaetoceros	*Chaetoceros costatus*	v9	-	1
					Chaetoceros curvisetus	v9	-	1
					Chaetoceros danicus	v4	1	-
					Chaetoceros debilis	v9	-	1
					Chaetoceros dichatoensis	v9	-	4
					Chaetoceros didymus	v9	-	1
					Chaetoceros lorenzianus	v4	1	-
					Chaetoceros muellerii	v9	-	1
					Chaetoceros rostratus	v9	-	1
					Chaetoceros socialis debilis	v9	-	1
					Chaetoceros tenuissimus	v9	-	1
			Leptocylindraceae	Leptocylindrus	*Leptocylindrus aporus*	v9	-	1
					Leptocylindrus convexus	v9	-	1
					Leptocylindrus danicus	v9	-	1
					Leptocylindrus minimus	v9	-	1
		Cymatosirales	Cymatosiraceae	Arcocellulus	*Arcocellulus cornucervis*	v9	-	1
				Brockmanniella	*Brockmanniella brockmannii*	v9	-	1
		Lithodesmiales	Lithodesmiaceae	Ditylum	*Ditylum brightwellii*	v9	-	1
		Thalassiosirales	Skeletonemaceae	Skeletonema	*Skeletonema marinoi*	v9	-	1
			Stephanodiscaceae	Cyclotella	*Cyclotella striata*	v9	-	1
			Thalassiosiraceae	Minidiscus	*Minidiscus trioculatus*	v9	-	1
				Thalassiosira	*Thalassiosira delicatula*	v9	-	1
					Thalassiosira gravida	v4	1	-
					Thalassiosira tenera	v9	-	1

GOLDEN/BROWN/OTHER								
Phylum	***class***	***order***	***family***	***genus***	***species***	***region***	***ASV v4***	***ASV v9***
Gyrista	Chrysophyceae	Paraphysomonadales	Paraphysomonadaceae	Paraphysomonas	*Paraphysomonas foraminifera*	v9	-	2
Gyrista	Dictyochophyceae	Dictyochophyceae X	Dictyochales	Dictyocha	*Dictyocha speculum*	v9	-	1
			Florenciellales	Pseudochattonella	*Pseudochattonella verruculosa*	v9	-	2
			Pedinellales	Pseudopedinella	*Pseudopedinella elastica*	v9	-	1
				Pteridomonas	*Pteridomonas danica*	v9	-	1

Phylum	*class*	*order*	*family*	*genus*	*species*	*region*	*ASV v4*	*ASV v9*
Gyrista	Raphidophyceae	Raphidophyceae X	Raphidophyceae XX	Fibrocapsa	*Fibrocapsa japonica*	v9	-	1

HAPTOPHYTES

Phylum	*class*	*order*	*family*	*genus*	*species*	*region*	*ASV v4*	*ASV v9*
Haptophyta	Prymnesiophyceae	Isochrysidales	Noelaerhabdaceae	Gephyrocapsa	*Gephyrocapsa oceanica*	v4, v9	2	2
		Phaeocystales	Phaeocystaceae	Phaeocystis	*Phaeocystis cordata*	v4, v9	2	1
					Phaeocystis globosa	v4, v9	3	1
					Phaeocystis jahnii	v9	-	1
		Prymnesiales	Prymnesiales X	Chrysocampanula	*Chrysocampanula spinifera*	v9	-	1
			Chrysochromulinaceae	Chrysochromulina	*Chrysochromulina simplex*	v9	-	4
		Prymnesiophyceae X	Braarudosphaeraceae	Braarudosphaera	*Braarudosphaera bigelowii*	v4, v9	3	1

Supplementary Table S2. List of ASVs identified to species level for tropical site samples (Tikioki, Rarotonga, Cook Islands), detected by the 18s rDNA V4 and V9 gene regions.

GREEN ALGAE

Phylum	*class*	*order*	*family*	*genus*	*species*	*region*	*ASV v4*	*ASV v9*
Chlorophyta	Chlorodendrophyceae	Chlorodendrales	Chlorodendraceae	Tetraselmis	*Tetraselmis_marina*	v9	-	1
	Chlorophyceae	Sphaeropleales	Sphaeropleales_X	Desmodesmus	*Desmodesmus_armatus*	v9	-	1
					Desmodesmus_opoliensis	v9	-	2
	Chloropicophyceae	Chloropicales	Chloropicaceae	Chloroparvula	*Chloroparvula_B2_sp.*	v9	-	2
					Chloroparvula_B3_sp.	v9	-	5
				Chloropicon	*Chloropicon_roscoffensis*	v9	-	1
					Chloropicon_sieburthii	v9	-	1
	Mamiellophyceae	Mamiellales	Mamiellaceae	Micromonas	*Micromonas_clade_B5*	v9	-	2
	Pedinophyceae	Marsupiomonadales	Marsupiomonadaceae	Marsupiomonas	*Marsupiomonas_pelliculata*	v9	-	1
	Pyramimonadophyceae	Pyramimonadales	Pyramimonadaceae	Pyramimonas	*Pyramimonas_gelidicola*	v9	-	1

DINOFLAGELLATES

Phylum	*class*	*order*	*family*	*genus*	*species*	*region*	*ASV v4*	*ASV v9*
Dinoflagellata	Dinophyceae	Dinophysiales	Oxyphysiaceae	Phalachroma	*Phalacroma_doryphorum*	v4	1	-
					Phalacroma_mitra	v4	2	-
					Phalacroma_porodictyum	v4	1	-
					Phalacroma_rotundatum	v4	1	-
		Gonyaulacales	Ceratiaceae	Tripos	*Tripos_furca*	v4	2	-
					Tripos_fusus	v9	-	1
					Tripos_tenuis	v9	-	1
			Gonyaulacaceae	Spiniferites	*Spiniferites_mirabilis*	v4	3	-
				Gonyaulax	*Gonyaulax_polygramma*	v4, v9	2	3
			Lingulodiniaceae	Lingulodinium	*Lingulodinium_polyedra*	v9	-	1
			Ostreopsidaceae	Centrodinium	*Centrodinium_punctatum*	v4	2	-
			Protoceratiaceae	Protoceratium	*Protoceratium_reticulatum*	v9	-	1
				Schuettiella	*Schuettiella_mitra*	v4	1	-
				Ceratocorys	*Ceratocorys_horrida*	v4, v9	1	1
			Pyrocystaceae	Alexandrium	*Alexandrium_insuetum*	v9	-	1
					Alexandrium_tamiyavanichii	v4	2	-
				Coolia	*Coolia_canariensis*	v4, v9	13	1
					Coolia_monotis	v4, v9	2	1

Phylum	***class***	***order***	***family***	***genus***	***species***	***region***	***ASV v4***	***ASV v9***
Dinoflagellata	Dinophyceae	Gonyaulacales	Pyrocystaceae	Fragilidium	*Fragilidium_mexicanum*	v4	2	-
				Fukuyoa	*Fukuyoa_yasumotoi*	v9	-	1
				Gambierdiscus	*Gambierdiscus_australes*	v4, v9	4	2
					Gambierdiscus_caribaeus	v9	-	1
					Gambierdiscus_carpenteri	v4, v9	5	1
					Gambierdiscus_honu	v4, v9	10	2
					Gambierdiscus_lapillus	v9	-	1
					Gambierdiscus_pacificus	v4	10	-
					Gambierdiscus_polynesiensis	v9	-	1
				Ostreopsis	*Ostreopsis_lenticularis*	v4, v9	3	5
					Ostreopsis_ovata	v4, v9	1	1
				Pyrocystis	*Pyrocystis_lunula*	v9	-	2
				Triadinium	*Triadinium_polyedricum*	v4, v9	1	2
		Gymnodiniales	Ceratoperidiniaceae	Ceratoperidinium	*Ceratoperidinium_falcatum*	v4	2	-
			Kareniaceae	Karlodinium	*Karlodinium_veneficum*	v9	-	1
			Gymnodiniaceae	Amphidinium	*Amphidinium_massartii*	v9	-	1
					Amphidinium_operculatum	v4	3	-
					Amphidinium_sp.	v4	17	2
					Amphidinium_stirisquamtum	v4	1	-
				Gymnodinium	*Gymnodinium_dorsalisulcum*	v4, v9	1	1
					Gymnodinium_sp.	v4	3	-
				Gyrodinium	*Gyrodinium_dominans*	v4	1	-
					Gyrodinium_fusiforme	v9	-	1
					Gyrodinium_gutrula	v9	-	1
					Gyrodinium_helveticum	v4	2	-
					Gyrodinium_jinhaense	v4	1	-
				Lepidodinium	*Lepidodinium_viride*	v9	-	1
				Margalefidinium	*Margalefidinium_fulvescens*	v9	-	3
					Margalefidinium_polykrikoides	v4, v9	2	1
				Moestrupia	*Moestrupia_oblonga*	v4	9	-
				Spiniferodinium	*Spiniferodinium_galeiforme*	v9	-	1
		Peridiniales	Blastodiniaceae	Blastodinium	*Blastodinium_contortum*	v4, v9	2	1
					Blastodinium_mangini	v4, v9	1	1
					Blastodinium_pruvoti	v4	1	-

Phylum	*class*	*order*	*family*	*genus*	*species*	*region*	*ASV v4*	*ASV v9*
Dinoflagellata	Dinophyceae	Peridiniales	Blastodiniaceae	Blastodinium	*Blastodinium_spinulosum*	v9	-	6
			Peridiniales_X	Bysmatrum	*Bysmatrum_granulosum*	v4	4	-
					Bysmatrum_subsalsum	v4	4	-
			Kryptoperidiniaceae	Dinothrix	*Dinothrix_paradoxa*	v9	-	1
				Durinskia	*Durinskia_agilis*	v4	1	-
					Durinskia_dybowskii	v4	3	-
			Protoperidiniaceae	Protoperidinium	*Protoperidinium_bipes*	v4	2	-
					Protoperidinium_crassipes	v4	1	-
					Protoperidinium_divergens	v9	-	1
					Protoperidinium_elegans	v4	2	-
					Protoperidinium_pellucidum	v4, v9	2	1
			Thoracosphaeraceae	Scrippsiella	*Scrippsiella_erinaceus*	v9	-	1
		Prorocentrales	Prorocentraceae	Prorocentrum	*Prorocentrum_concavum*	v4	1	-
					Prorocentrum_cordatum	v9	-	1
					Prorocentrum_emarginatum	v4, v9	7	2
					Prorocentrum_fukuyoi	v4, v9	10	5
					Prorocentrum_hoffmannianum	v9	-	2
					Prorocentrum_lima	v4	2	-
					Prorocentrum_nanum	v9	-	1
					Prorocentrum_sipadanense	v9	-	1
		Suessiales	Suessiaceae	Biecheleria	*Biecheleria_cincta*	v4	2	-
				Pelagodinium	*Pelagodinium_beii*	v4	2	-
				Polarella	*Polarella_glacialis*	v4	1	-
			Symbiodiniaceae	Cladocopium	*Cladocopium_goreaui*	v9	-	4
		Torodiniales	Torodiniaceae	Torodinium	*Torodinium_robustum*	v4	2	-
					Torodinium_teredo	v9	-	1

PENNATE DIATOMS

Phylum	*class*	*order*	*family*	*genus*	*species*	*region*	*ASV v4*	*ASV v9*
Gyrista	Bacillariophyceae	Bacillariales	Bacillariaceae	Fragilariopsis	*Fragilariopsis_sublineata*	v9	-	1
				Pseudo-nitzschia	*Pseudo-nitzschia_delicatissima*	v9	-	1
		Cymbellales	Cymbellaceae	Cymbella	*Cymbella_cistuliformis*	v9	-	1
		Fragilariales	Fragilariaceae	Grammonema	*Grammonema_striatula*	v9	-	1
		Licmophorales	Licmophoraceae	Licmophora	*Licmophora_flabellata*	v9	-	1
		Naviculales	Naviculaceae	Haslea	*Haslea_avium*	v9	-	1

Phylum	*class*	*order*	*family*	*genus*	*species*	*region*	*ASV v4*	*ASV v9*
Gyrista	Bacillariophyceae	Naviculales	Naviculaceae	Navicula	*Navicula cryptocephala var. veneta*	v9	-	1
					Navicula_perminuta	v9	-	1
					Navicula_tripunctata	v9	-	1
		Rhabdonematales	Tabellariaceae	Asterionella	*Asterionella_glacialis*	v4	1	-
			Grammatophoraceae	Hyalosira	*Hyalosira_delicatula*	v4	1	-
		Rhopalodiales	Rhopalodiaceae	Epithemia	*Epithemia_catenata*	v4	1	-
					Epithemia_pelagica	v9	-	1
**RADIAL CENTRIC DIATOMS**								
Phylum	*class*	*order*	*family*	*genus*	*species*	*region*	*ASV v4*	*ASV v9*
Gyrista	Coscinodiscophyceae	Coscinodiscales	Hemidiscaceae	Actinocyclus	*Actinocyclus_curvatulus*	v9	-	1
		Melosirales	Hyalodiscaceae	Hyalodiscus	*Hyalodiscus_scoticus*	v9	-	1
		Stellarimales	Stellarimaceae	Stellarima	*Stellarima_microtrias*	v9	-	1
**POLAR CENTRIC DIATOMS**								
Phylum	*class*	*order*	*family*	*genus*	*species*	*region*	*ASV v4*	*ASV v9*
Gyrista	Mediophyceae	Ardissoneales	Ardissoneaceae	Climacosphenia	*Climacosphenia_moniligera*	v9	-	1
		Chaetocerotales	Chaetocerotaceae	Chaetoceros	*Chaetoceros_tenuissimus*	v9	-	1
		Hemiaulales	Hemiaulaceae	Hemiaulus	*Hemiaulus_sinensis*	v9	-	1
		Lithodesmiales	Lithodesmiaceae	Lithodesmium	*Lithodesmium_undulatum*	v9	-	1
		Probosciales	Probosciaceae	Proboscia	*Proboscia_alata*	v9	-	1
		Thalassiosirales	Thalassiosiraceae	Porosira	*Porosira_glacialis*	v9	-	1
				Thalassiosira	*Thalassiosira_tumida*	v9	-	1
**GOLDEN/BROWN/OTHER**								
Phylum	*class*	*order*	*family*	*genus*	*species*	*region*	*ASV v4*	*ASV v9*
Gyrista	Bolidophyceae	Parmales	Triparmaceae	Triparma	*Triparma_eleuthera*	v9	-	1
	Dictyochophyceae	Dictyochophyceae_X	Rhizochromulinales	Rhizochromulina	*Rhizochromulina_marina*	v9	-	1
	Pinguiophyceae	Pinguiochrysidales	Pinguiochrysidaceae	Pinguiochrysis	*Pinguiochrysis_pyriformis*	v9	-	1
				Pinguiococcus	*Pinguiococcus_pyrenoidosus*	v9	-	1
	Raphidophyceae	Raphidophyceae_X	Raphidophyceae_XX	Haramonas	*Haramonas_dimorpha*	v9	-	1
**HAPTOPHYTES**								
Phylum	*class*	*order*	*family*	*genus*	*species*	*region*	*ASV v4*	*ASV v9*
Haptophyta	Pavlovophyceae	Pavlovales	Pavlovaceae	Exanthemachrysis	*Exanthemachrysis_gayraliae*	v9	-	1
	Prymnesiophyceae	Prymnesiales	Chrysochromulinaceae	Chrysochromulina	*Chrysochromulina_campanulifera*	v9	-	1

Phylum	***class***	***order***	***family***	***genus***	***species***	***region***	***ASV v4***	***ASV v9***
	Prymnesiophyceae	Prymnesiales	Chrysochromulinaceae	Chrysochromulina	*Chrysochromulina_leadbeateri*	v9	-	2
					Chrysochromulina_throndsenii	v9	-	1
			Prymnesiaceae	Prymnesium	*Prymnesium_parvum*	v9	-	1
		Prymnesiophyceae_X	Braarudosphaeraceae	Braarudosphaera	*Braarudosphaera_bigelowii*	v9	-	1

Supplementary table S3: Temperate site sample and replicate information for metabarcoding and cell counts.

Site	*Point*	*Replicate*	*Filtered Vol. (mL; metabarcoding)*	*DNA conc. (ng/µl)*	*Cell count volume*	*Site*	*Point*	*Replicate*	*Filtered Vol. (mL; metabarcoding)*	*DNA conc. (ng/µl)*	*Cell count volume*
Temperate (N1)	A	1	100 – 200	57.1	10	Tropical (T3)	A	1	100 – 200	13.4	10
		2	100 – 200	59.4				2	100 – 200	10.35	
		3	100 – 200	31.5				3	100 – 200	5.1	
	B	1	100 – 200	50.2	10		B	1	100 – 200	8.15	10
		2	100 – 200	37.6				2	100 – 200	8.35	
		3	100 – 200	38.2				3	100 – 200	6.35	
	C	1	100 – 200	24.4	10		C	1	100 – 200	5.2	10
		2	100 – 200	34.8				2	100 – 200	8.85	
		3	100 – 200	48.5				3	100 – 200	7.5	
	D	1	100 – 200	45.7	10		D	1	100 – 200	8.1	10
		2	100 – 200	34.2				2	100 – 200	5.4	
		3	100 – 200	38.8				3	100 – 200	11.2	
	E	1	100 – 200	40.9	10		E	1	100 – 200	8.0	10
		2	100 – 200	34.3				2	100 – 200	6.3	
		3	100 – 200	51.8				3	100 – 200	5.8	
	F	1	100 – 200	28.2	10		F	1	100 – 200	5.05	10
		2	100 – 200	48.3				2	100 – 200	11.1	
		3	100 – 200	27.8				3	100 – 200	10.8	
	G	1	100 – 200	31.6	10		G	1	100 – 200	11.3	10
		2	100 – 200	51.5				2	100 – 200	7.0	
		3	100 – 200	42.1				3	100 – 200	9.0	
	H	1	100 – 200	40.4	10		H	1	100 – 200	10.9	10
		2	100 – 200	38.9				2	100 – 200	12.4	
		3	100 – 200	44.3				3	100 – 200	10.25	
	I	1	100 – 200	37.1	10		I	1	100 – 200	8.3	10
		2	100 – 200	41.9				2	100 – 200	9.3	
		3	100 – 200	46.1				3	100 – 200	5.65	

Supplementary Table S4. Primer Sequences, target region and reaction conditions used for metabarcoding.

	Primer sequences (3'-5')	Target	Thermocycling conditions	References
Uni18S-F	AGG GCA AKY CTG GTG CCA GC	V4 region, 18S rDNA	95 °C for 5 m<br>30x : 95 °C for 30 s, 54 °C for 30 s + 72 °C for 45 s<br>Final ext: 72 °C for 7 m	[1]
Uni18S-R	GRC GGT ATC TRA TCG YCT T			
1380F	CCC TGC CHT TTG TAC ACA C	V9 region, 18S rDNA	94 °C for 3 m<br>30x: 94 °C for 30 s, 57 °C for 1 m + 72 °C for 1.5 m<br>Final ext: 72 °C for 10 m	[2]
1510R	CCT TCY GCA GGT TCA CCT AC			

References

1. Zhan, A., et al., *High sensitivity of 454 pyrosequencing for detection of rare species in aquatic communities.* Methods Ecol. Evol., 2013. **4**(6): p. 558-565.
2. Amaral-Zettler, L.A., et al., *A method for studying protistan diversity using massively parallel sequencing of V9 hypervariable regions of small-subunit ribosomal RNA genes.* PLoS One, 2009. **4**(7): p. e6372.

Supplementary table S5: Summary of initial sequencing reads and those remaining after each step of the DADA2 pipeline.

		18S V4 REGION							18S V9 REGION						
Sample Names	ecoregion	input	filtered	Denoise Forward	Denoise Reverse	merged	clean	%	input	filtered	Denoise Forward	Denoise Reverse	merged	clean	%
N1a1	temperate	75755	61413	60931	60900	57098	49858	66	259311	252015	241158	238865	230795	222694	86
N1a2	temperate	43144	35218	34973	34836	33355	28483	66	102842	100832	97748	96759	94169	90738	88
N1a3	temperate	59093	48192	47692	47660	44875	39846	67	122462	119901	115714	114438	110717	107919	88
N1b1	temperate	32913	26125	25965	25900	24572	20810	63	102580	100509	96757	95872	93097	88094	86
N1b2	temperate	35093	28969	28781	28731	27462	23306	66	85726	83779	80374	79509	77160	73451	86
N1b3	temperate	38855	31627	31472	31411	29938	25300	65	100318	97838	93642	92023	89028	84638	84
N1c1	temperate	63616	51092	50827	50654	48575	42114	66	164602	160566	154417	153245	148746	142332	86
N1c2	temperate	18235	14732	14618	14619	13818	10705	59	127686	124702	120225	117840	114535	109551	86
N1c3	temperate	22729	18400	18254	18216	17218	13830	61	142090	138130	131943	130355	125662	120992	85
N1d1	temperate	32348	26517	26321	26251	25054	20680	64	83356	81181	77918	77207	74427	71568	86
N1d2	temperate	41586	34075	33933	33795	32189	27200	65	89776	87501	83856	82859	80176	76405	85
N1d3	temperate	99394	81530	81147	80947	77839	67210	68	130727	127911	123378	121547	118310	113665	87
N1e1	temperate	70528	57685	57407	57301	55101	47732	68	313077	304284	291269	289399	279682	271158	87
N1e2	temperate	60175	49324	49069	49035	47163	40973	68	94084	92055	88924	88544	86009	83347	89
N1e3	temperate	26543	21774	21632	21578	20607	16772	63	98104	95946	92532	91871	89004	85151	87
N1f1	temperate	64184	51966	51681	51499	49104	42581	66	159245	154567	148432	147266	142932	136061	85
N1f2	temperate	56348	46853	46630	46485	44473	37686	67	67808	65936	63405	62783	60750	56680	84
N1f3	temperate	51763	42519	42308	42205	40599	34645	67	159435	155432	149902	147563	143410	137226	86
N1g1	temperate	57747	46354	46048	45967	43840	37556	65	148035	144514	138689	137780	133405	128634	87
N1g2	temperate	14154	11475	11393	11352	10810	7720	55	125868	122057	117948	115778	112603	106371	85
N1g3	temperate	50205	41573	41337	41326	39393	32722	65	98812	95315	90833	89969	86549	80423	81
N1h1	temperate	32482	26846	26663	26573	25107	20844	64	112292	108809	104700	103826	100298	91373	81
N1h2	temperate	50285	41797	41591	41491	40074	33086	66	103329	100472	96591	95703	92744	87430	85
N1h3	temperate	62738	51661	51419	51338	49156	41251	66	85430	83187	80103	78947	76540	71300	83
N1i1	temperate	82124	68674	68274	68206	65069	55463	68	210215	205256	200101	197582	193090	182940	87
N1i2	temperate	53519	45136	44962	44853	43377	36230	68	84495	83112	81641	80710	79174	75749	90
N1i3	temperate	77053	65603	65328	65156	62614	52957	69	79973	78368	76682	75378	73808	69791	87
ExtBL1	blank	3588	856	842	844	745	0	0	1411	1281	1052	948	767	0	0
PCRBL1	blank	6200	2169	2162	2157	2087	0	0	2129	1918	1596	1502	1303	0	0
H20BL1	blank	761	172	159	155	147	0	0	1484	1360	1060	994	812	0	0

Sample Names	ecoregion	18S V4 REGION input	filtered	denoise Forward	Denoise Reverse	merged	clean	%	18S V9 REGION input	filtered	denoise Forward	Denoise Reverse	merged	clean	%
T3a1	tropical	50949	31010	30636	30531	27605	24460	48	209371	202996	196518	195589	190685	170060	81
T3a2	tropical	112068	67652	67168	66971	61199	54041	48	39190	37789	36592	35786	34879	33033	84
T3a3	tropical	146794	88240	87688	87460	82749	78714	54	108175	104920	101763	100870	98438	95504	88
T3b1	tropical	145243	91539	90936	90850	86630	73118	50	49440	48101	46677	45128	43986	42380	86
T3b2	tropical	78903	45784	45453	45322	42717	37016	47	91504	89474	87126	84551	82091	79445	87
T3b3	tropical	76364	45674	45281	45182	42877	39370	52	68839	66826	64388	63488	61633	59059	86
T3c1	tropical	64594	35918	35611	35530	33509	30081	47	71248	66060	63626	62058	60411	57602	81
T3c2	tropical	62684	34253	33893	33835	31546	29139	46	76505	64059	62044	59555	57968	46476	61
T3c3	tropical	41027	22049	21742	21616	20107	18545	45	44047	42919	41618	39294	38014	36204	82
T3d1	tropical	58760	33367	33027	32941	31418	28448	48	52587	51177	49555	49262	47961	46130	88
T3d2	tropical	368	25	7	11	0	0	0	91406	89588	87626	87359	85385	82686	90
T3d3	tropical	341952	189164	188269	187922	179299	150910	44	44922	43459	41892	41565	40264	35996	80
T3e1	tropical	34059	18884	18658	18558	17266	15488	45	115959	111874	107440	107895	104370	101255	87
T3e2	tropical	66308	37753	37399	37299	34272	31946	48	92314	88145	85750	84396	82402	77271	84
T3e3	tropical	51425	26869	26632	26497	24744	21894	43	84191	80988	78297	77832	75703	62065	74
T3f1	tropical	64454	38957	38655	38498	36428	33505	52	59476	57676	55943	54765	53327	50978	86
T3f2	tropical	89590	51350	50886	50830	47566	41507	46	94981	92220	89007	88326	85511	82467	87
T3f3	tropical	133138	77127	76593	76401	69629	62817	47	75214	71001	68574	67718	65829	63488	84
T3g1	tropical	49314	28796	28460	28391	26682	24361	49	34525	32629	29701	29660	27910	26871	78
T3g2	tropical	16870	10159	10022	9979	9487	9011	53	68512	67130	65532	64053	62443	60217	88
T3g3	tropical	33870	20060	19824	19804	18691	17763	52	41177	39621	37440	36371	34923	32697	79
T3h1	tropical	46672	28265	27938	27882	26027	25005	54	42607	41000	39169	39841	38204	36477	86
T3h2	tropical	33	1	1	1	0	0	0	110309	106817	102968	104762	100879	96822	88
T3h3	tropical	82453	48411	48002	47837	45562	41639	51	47217	45660	43708	44009	42512	40426	86
T3i1	tropical	102	3	1	1	0	0	0	111171	104089	99699	101450	97176	90691	82
T3i2	tropical	66206	37331	37098	36973	34677	32017	48	54043	52072	50302	49529	48479	37332	69
T3i3	tropical	34665	20159	20002	19892	19123	17886	52	95837	92949	89966	90244	87898	84858	89
EXT_BL6	blank	148	11	1	3	0	0	0	163	124	57	64	34	0	0
PCR_BL3	blank	469	118	116	96	71	0	0	210	167	71	55	32	0	0
H20_BL3	blank	131	4	2	1	1	0	0	363	295	131	117	65	0	0

Supplementary table S6: ASV designations of all sequences from the metabarcoding results of 18s V4 and V9 regions.

Division	V9 ASVs	V9 Proportion	V4 ASVs	V4 Proportion
Unclassified	22	1%	0	0%
Bacteria	0	0%	0	0%
Eukaryote (excluding microalgae)	3420	81%	1838	70%
Eukaryotic microalgae	803	19%	795	30%
Total	4245	100%	2633	100%

Supplementary table S7: Alpha diversity values based on metabarcoding sequences from 18s V4 and V9 regions at tropical and temperate site.

				18S V4 REGION			18S V9 REGION		
**Site ID**	**Sample point**	**ecoregion**	**Rarefy depth**	**Chao1**	**Shannon**	**InvSimpson**	**Chao1**	**Shannon**	**InvSimpson**
N1	a	Temperate	11000	173.2	3.2	9.2	270.4	3.5	9.7
N1	b	Temperate	11000	143.6	2.8	5.7	228.3	2.9	4.5
N1	c	Temperate	11000	151.9	2.8	6	231.8	3.1	5.4
N1	d	Temperate	11000	145.4	3.0	7.2	234.2	3.1	5.3
N1	e	Temperate	11000	150.5	2.8	5.9	247.6	3.0	5.2
N1	f	Temperate	11000	158.5	3.0	7.5	229.2	3.1	6.0
N1	g	Temperate	11000	129.1	2.7	5.7	232.5	3.0	5.1
N1	h	Temperate	11000	-	-	-	218.5	3.5	10.3
N1	i	Temperate	11000	159.3	2.9	6.9	228.5	3.4	8.7
T3	a	Tropical	11000	216.1	3.5	14.4	238.9	2.6	5.1
T3	b	Tropical	11000	183.6	2.9	8.6	210.0	2.3	4.4
T3	c	Tropical	11000	168.5	3.1	10	192.1	2.3	4.4
T3	d	Tropical	11000	183.0	2.8	7.6	196.5	2.1	3.9
T3	e	Tropical	11000	170.2	2.8	7.9	199.7	1.9	3.2
T3	f	Tropical	11000	172.0.	2.9	8.7	204.8	2.0	3.5
T3	g	Tropical	11000	145.6	3.3	13	186.1	2.5	5.1
T3	h	Tropical	11000	150.0	2.9	7.9	189.7	2.1	3.8
T3	i	Tropical	11000	117.3	2.9	8.1	201.2	2.1	3.6
